# Dexmedetomidine improves septic acute kidney injury by inhibiting inflammation and oxidative stress through the activation of the Pink1/Park2 autophagy pathway

**DOI:** 10.1080/0886022X.2025.2513677

**Published:** 2025-06-08

**Authors:** Qiuxia Liao, Zhi Feng, Hairong Lin, Ye Zhou, Xinxin Lin, Xiao Lin, Huichang Zhuo

**Affiliations:** a Department of Intensive Care Unit, First Affiliated Hospital of Fujian Medical University, Fuzhou, Fujian, China; b Department of Intensive Care Unit, National Regional Medical Center, Binhai Campus of the First Affiliated Hospital, Fuzhou, Fujian, China; c Department of Thoracic Surgery, First Affiliated Hospital of Fujian Medical University, Fuzhou, Fujian, China

**Keywords:** Dexmedetomidine, Pink1, Park2, autophagy, sepsis, acute kidney injury

## Abstract

Impaired autophagy is a key factor in the development of septic acute kidney injury (SAKI). Dexmedetomidine—an α_2_ adrenergic agonist widely used as a sedative—exerts protective effects in SAKI. However, its correlation with autophagy remains unclear. Consequently, this study aimed to investigate whether the protective effect of dexmedetomidine against SAKI is related to the Pink1/Park2 autophagy pathway. Dexmedetomidine was intraperitonally administered to mice before inducing SAKI with lipopolysaccharide. Subsequently, kidney structure, inflammatory markers, renal function, oxidative stress levels, mitochondrial 16S rRNA expression, autophagy-related protein levels (Pink1, Park2, and Optineurin), and renal cell apoptosis rates were evaluated. Dexmedetomidine reduced inflammatory factors, such as tumor necrosis factor-α, interleukin (IL)-18, IL-6, and IL-1β, and improved kidney function by decreasing serum cystatin C, creatinine, blood urea nitrogen, kidney injury molecule-1, and neutrophil gelatinase-associated lipocalin. Furthermore, it also alleviated kidney tissue damage. Additionally, dexmedetomidine enhanced mitochondrial function; reduced kidney tissue levels of reactive oxygen species, catalase, malondialdehyde, and glutathione; increased superoxide dismutase activity; upregulated mt16S expression; promoted the expression of autophagy-related proteins (Pink1, Park2, and Optineurin); and reduced renal cell apoptosis rates. Notably, all results were statistically significant. Overall, our findings revealed that dexmedetomidine may mitigate inflammation, oxidative stress, and renal dysfunction in mice with SAKI by upregulating the Pink1/Park2-mediated autophagy pathway. These preliminary findings highlight dexmedetomidine’s potential role in SAKI management and warrant further validation in large scale studies.

## Introduction

Sepsis is a major cause of multiple organ dysfunction and is frequently associated with acute kidney injury, referred to as septic acute kidney injury (SAKI) [1–4]. Globally, approximately 19 million cases of sepsis occur each year, with one-third of patients developing acute kidney injury [5,6]. In intensive care unit (ICU) settings, the incidence of SAKI rises to 40%–50% [7]. SAKI is characterized by rapid progression, poor prognosis, and a high mortality rate of up to 22%–24% [1,3], along with an increased risk of chronic complications [8]. Despite its clinical significance, effective preventive or therapeutic interventions for SAKI remain limited, largely due to the complex and incompletely understood pathophysiological mechanisms. Recent evidence highlights the pivotal role of energy metabolism disturbances in SAKI, particularly those involving mitochondrial dysfunction [9].

Mitochondria are central regulators of cellular energy homeostasis, and their injury contributes to oxidative stress, inflammation, and apoptosis in SAKI. Autophagy, a cellular process that eliminates damaged or dysfunctional mitochondria, plays a key role in maintaining mitochondrial quality and cellular integrity [10]. The PTEN-induced putative kinase 1 (Pink1)/Parkin RBR E3 ubiquitin-protein ligase (Park2) pathway is a well-established regulator of mitophagy and has been implicated in the pathogenesis of SAKI [11,12]. Upon mitochondrial depolarization, Pink1 accumulates on the outer mitochondrial membrane and recruits Park2, which facilitates the ubiquitination of outer membrane proteins and initiates mitophagosome formation. Optineurin, a ubiquitin-binding autophagy adaptor, acts downstream of Park2 and contributes to mitophagy by recognizing ubiquitinated mitochondria and mediating their delivery to autophagosomes *via* interaction with LC3.

Dexmedetomidine, an α_2_-adrenergic receptor agonist widely used as a sedative in clinical settings, has been shown to improve systemic and renal hemodynamics, reduce norepinephrine release, and exert anti-inflammatory effects [13]. Several experimental and clinical studies have suggested its protective potential in SAKI [14]; however, the molecular mechanisms underlying this protection remain unclear. In particular, whether the protective effects of dexmedetomidine are mediated through the regulation of mitophagy has not been thoroughly investigated.

Therefore, the aim of this study was to evaluate whether dexmedetomidine could exert renoprotective effects in an LPS-induced murine model of SAKI by modulating the Pink1/Park2/Optineurin-dependent mitophagy pathway. Additionally, we assessed its potential impact on inflammation, oxidative stress and renal cell apoptosis to explore the potential mechanistic basis for its therapeutic effects.

## Materials and methods

### Construction of a mouse model of SAKI

Nine male mice, approximately 20 g in weight and 8 weeks old, were purchased from Zhuhai Baisitong Biotechnology Co., Ltd. (Zhuhai, China) and randomly assigned to three groups: the control group, the group with SAKI induced using lipopolysaccharide (LPS group), and the group with SAKI treated using dexmedetomidine (LPS+DEX group). Mice in the LPS+DEX group received an intraperitoneal injection of 200 µg/kg of dexmedetomidine hydrochloride (D129813, Aladdin, Shanghai, China) 30 min before modeling, whereas the control and LPS groups received an equivalent volume of 0.9% sodium chloride (NaCl) solution intraperitoneally. Thereafter, mice in the LPS and LPS+DEX groups were administered LPS (LPS, ST1470, Biyuntian, Shanghai) intraperitoneally at a dose of 20 mg/kg 30 min after dexmedetomidine administration to induce SAKI. The control group received an intraperitoneal injection of the same volume of 0.9% NaCl solution. Four hours after LPS injection, the mice were anesthetized with 2% pentobarbital, and blood samples and kidney tissues were collected for further analysis.

### Ethical considerations

Before the experiments, the mice were housed in specific pathogen-free laboratory cages for 7 days. The indoor temperature was maintained at 22 °C with relative humidity at 50 ± 10% and a 12-h light/dark cycle. Food and water were provided *ad libitum*. All animal experiments were approved by the Experimental Animal Center of Fujian Medical University (Ethics Number: IACUC FJMU2024-0407), and efforts were made to minimize animal injury and suffering.

### Measurement of inflammatory cytokines and kidney function

After blood collection, the samples were allowed to stand undisturbed at room temperature for 30–60 min to allow natural coagulation and complete serum separation. This allowed, clear serum to be released from the blood clot, essential for minimizing the risk of hemolysis. Then, the samples were centrifuged at 3000 rpm for 10 min, and the supernatant was collected and stored at −80 °C for subsequent analysis.

The assays for inflammatory cytokines and renal function markers were based on enzyme-linked immunosorbent assay (ELISA) using a colorimetric reaction. The absorbance was measured at 450 nm, and the concentrations of each biomarker were calculated from the standard curves provided with the kits (Xinyu, Shanghai, China). Tumor necrosis factor α (TNF-α, XYM901321), interleukin (IL)-18 (IL-18, XYM901691), IL-6 (XYM901631), and (IL-1β) XYM900401) were measured to assess the inflammatory levels in mice. Cystatin C (Cys-C, XYM907801), creatinine (Cr, XYM906931), blood urea nitrogen (BUN, XYM906921), kidney injury molecule-1 (KIM1, XYM903181), and neutrophil gelatinase-associated lipocalin (NGAL, XYM902671) levels were measured to evaluate kidney function.

### Assessment of oxidative stress levels in kidney tissue

Mouse kidney tissue was collected, and an extraction buffer was added before centrifugation. Following centrifugation, the supernatant was obtained, and the levels of reduced glutathione (GSH, A006-2-1, Jiancheng, Nanjing), superoxide dismutase (SOD, BC5165, Sigma-Aldrich) activity, malondialdehyde (MDA, XYM908971, Xinyu, Shanghai), and catalase (CAT, XYM9441251, Xinyu, Shanghai) were measured following the manufacturer’s instructions.

### Reactive oxygen species (ROS) measurement

Kidney tissue was cut into small pieces, digested with a mixed enzyme digestion solution, and filtered using a nylon mesh to remove the tissue clumps. The filtered cells were collected and centrifuged. The supernatant was discarded, and the concentration of the cells was adjusted to approximately 1 × 10^6^ cells/mL to prepare a cell suspension. Dichlorodihydrofluorescein diacetate staining solution (S0033, BestBio) was added to the cell suspension and incubated in a cell culture incubator at 37 °C for 20 min. The supernatant was discarded, and the cell pellet was resuspended in Dulbecco’s Modified Eagle Medium (SH30243.01, HyClone). Finally, the samples were subjected to flow cytometry (NovoCyte D2060R, Agilent Technologies) to detect the fluorescence intensity and determine ROS levels.

### Renal tissue pathological analysis

The kidney tissues were fixed in formaldehyde and embedded in paraffin. Paraffin-embedded kidney tissues were then sliced in 4-µm-thick sections using a microtome. Subsequently, hematoxylin and eosin (G1004, Google Bio) staining was performed, and target areas were identified and photographed using an optical microscope.

### Terminal deoxynucleotidyl transferase dUTP nick-end labeling (TUNEL) staining for detecting cell apoptosis

Apoptosis was detected using the one-step TUNEL Apoptosis Detection Kit-CY3 (C1090, Biyuntian). Paraffin-embedded sections were placed in xylene (1330-20-7, Tianjin Fuyu Fine Chemicals Co., Ltd.) and deparaffinized twice for 5 min each. Sections were then rehydrated by sequential immersion in absolute ethanol (SJ0002, Weining) for 5 min, 90% ethanol for 2 min, 70% ethanol for 2 min, and distilled water for 2 min. Next, 20 μg/mL proteinase K was added for digestion, and the sections were incubated at 37 °C for 30 min, followed by washing with phosphate-buffered saline (PBS). Subsequently, 50 μL of TUNEL working solution was applied for color development, and the sections were incubated in the dark at 37 °C for 60 min. Thereafter, 4′,6-diamidino-2-phenylindole (DAPI) staining solution was added to stain the nuclei for 10 min, and the sections were washed twice with PBS for 5 min each. Finally, 20 μL of the anti-fade mounting medium was added, coverslips were applied, and the slides were photographed under a fluorescence microscope (Carl Zeiss, LSM800).

### Western blotting

Western blotting was performed to detect the expression levels of Pink1, Park2, and Optineurin in kidney tissues. Optineurin was included in our study to serve as an additional indicator of mitophagy activation. Briefly, kidney tissue samples (100 mg) were homogenized in lysis buffer, centrifuged, and the supernatant was boiled for 10 min. Total protein concentration was determined using a BCA protein assay. Approximately 50 µg of total protein per sample was loaded into each well, along with 3 µL of protein marker (26616, Thermo), and separated by SDS-polyacrylamide gel electrophoresis. The protein bands were then transferred to a polyvinylidene fluoride (PVDF) membrane (ISEQ00010, Merck) and blocked with a commercial blocking solution (Yili). The membranes were then incubated with primary antibodies against Pink1 (23274-1-AP, Proteintech), Park2 (14060-1-AP, Proteintech), and Optineurin (ab213556, Abcam) on a shaker at room temperature for 2 h, followed by incubation with secondary antibodies on a shaker at room temperature for 1 h. After washing, the BeyoECL Moon chemiluminescent detection reagent (P0018F, Biyuntian) was applied to the target bands on the membrane, and the membrane was developed using a chemiluminescence imaging system (Tanon-5200, Tanon). Finally, the levels of the target proteins were quantified and analyzed using Quantity One software (Bio-Rad Laboratories Inc., Hercules, CA, USA).

### Quantitative polymerase chain reaction (qPCR)

qPCR was performed using the specific mouse primers listed below: β-actin: Forward-5′-GATGCTGACCCTCATCCACT-3′′, Reverse-5′-TGAAGAGTTTTGGCGATGG-3′′ and mt16S: Forward-5′-ATTCCAATTCTCCAGGCATACG-3′′, Reverse-5′-GGGGTTCTTGTTTGCCGAGTT-3′′. The reaction mixture comprised 10 μL of ChamQ Universal SYBR qPCR (Q311-03, Vazyme), 1.5 μL of the forward primer, 1.5 μL of the reverse primer, 1 μL of cDNA template, and ddH2O as appropriate to make a final volume of 15 μL. PCR was performed using a real-time fluorescence qPCR instrument (Bio-Rad, CFX96), followed by data analysis.

### Statistical analysis

Statistical analyses were conducted using SPSS version 22.0. All data are presented as the mean ± standard deviation. A t-test was performed to determine statistically significant differences between the two groups. Statistical significance was set at *p* < 0.05.

## Results

### Dexmedetomidine reduces inflammatory cytokine production in mice with SAKI

**Figure 1.**
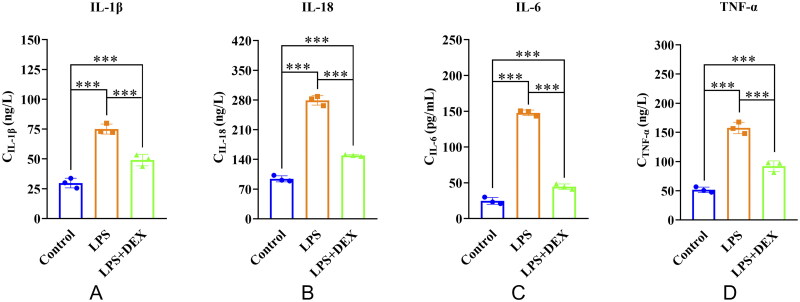
Dexmedetomidine reduces the production of inflammatory cytokines in mice with SAKI. A: Interleukin-1β (IL-1β); B: IL-18; C: IL-6; D: Tumor necrosis factor-α (TNF-α). ***Indicates *p* < 0.001 compared between the two groups.

The effects of dexmedetomidine on inflammatory cytokine production in mice with SAKI were evaluated. Compared with the control group, levels of IL-1β, IL-18, IL-6, and TNF-α were elevated in both the LPS and LPS+DEX groups. However, the levels of these cytokines were reduced in the LPS+DEX group compared with those in the LPS group, indicating dexmedetomidine-mediated reduction in inflammatory cytokine production in mice with SAKI (Figure 1).

### Dexmedetomidine reduces SAKI

**Figure 2.**
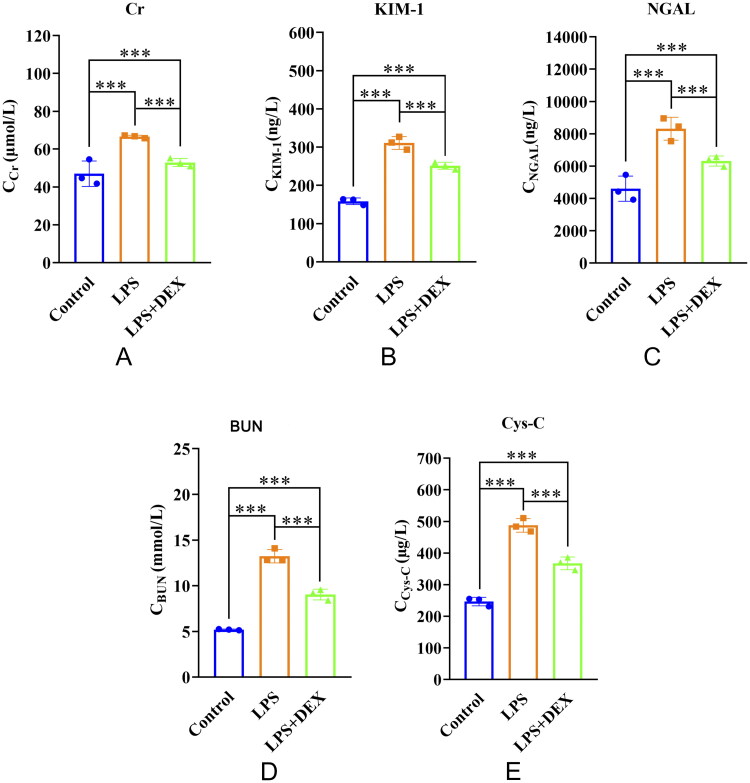
Dexmedetomidine alleviates SAKI. Levels of A: creatinine (Cr); B: kidney injury molecule-1 (KIM1); C: neutrophil gelatinase-associated lipocalin (NGAL); D: blood urea nitrogen (BUN); and E: cystatin C (Cys-C). ***Indicates a highly significant difference between the two groups (*p* < 0.001); **indicates a significant difference between the two groups (0.001<*p* < 0.01).

The effect of dexmedetomidine on kidney function was analyzed in mice with SAKI. Compared with the control group, the LPS and LPS+DEX groups exhibited increased levels of Cr, KIM1, NGAL, BUN, and Cys-C. However, the LPS+DEX group exhibited significantly lower levels of these markers than the LPS group, indicating that dexmedetomidine could mitigate SAKI (Figure 2).

### Dexmedetomidine reduces mitochondrial oxidative stress in mice with SAKI

**Figure 3.**
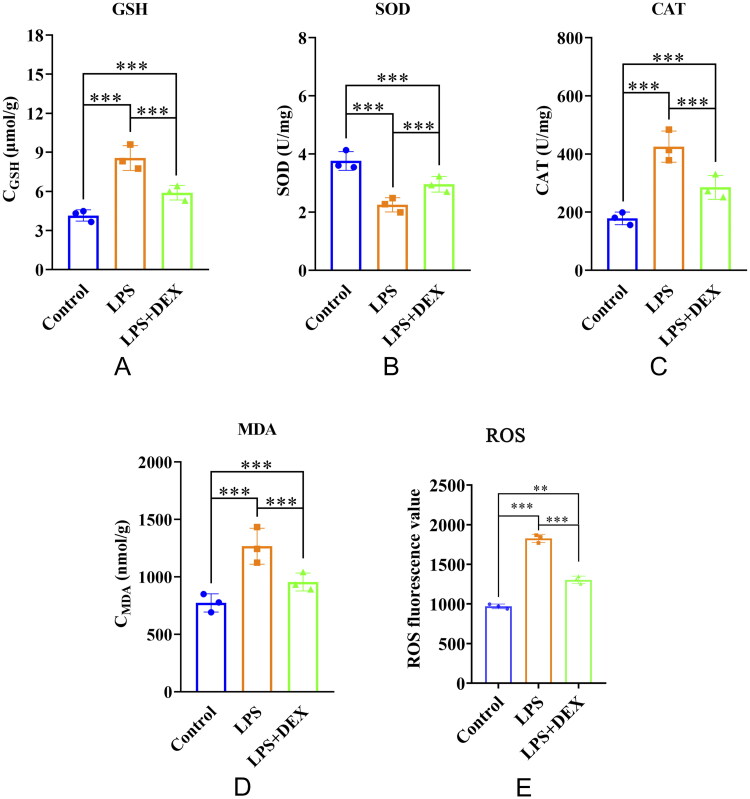
Dexmedetomidine reduces mitochondrial oxidative stress in mice with SAKI. A: Reduced levels of glutathione (GSH); B: superoxide dismutase (SOD) activity; C: catalase (CAT);D: malondialdehyde (MDA); E: reactive oxygen species (ROS). ***Indicates a comparison between the two groups (*p* < 0.001).

Levels of ROS, CAT, MDA, and GSH in kidney tissues were measured to evaluate the effect of dexmedetomidine on oxidative stress in mice with SAKI. Compared with the control group, the LPS group exhibited increased renal levels of GSH, MDA, CAT, and ROS, along with a decrease in SOD activities. Furthermore, compared with mice in the LPS group, those in the LPS+DEX group exhibited lower levels of GSH, MDA, CAT, and ROS in their kidney tissues, whereas SOD activity was significantly higher. Therefore, dexmedetomidine could reduce oxidative stress and protect mitochondrial function in SAKI (Figure 3).

### Dexmedetomidine increases mitochondrial 16S rRNA (mt16S) levels in mice with SAKI

**Figure 4.**
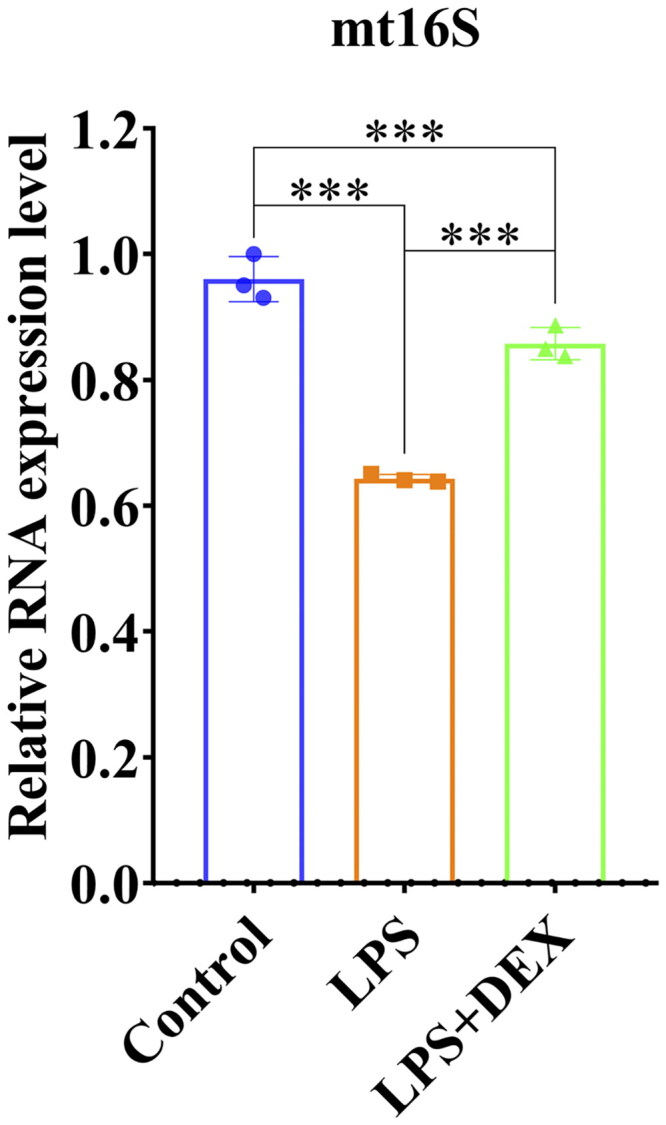
Dexmedetomidine elevated mt16S expression. ***Indicates *p* < 0.001, reflecting a highly significant difference.

Compared with the control group, the LPS group exhibited a significant decrease in mitochondrial mt16S levels. However, the LPS+DEX group exhibited a significant increase in its levels compared with those in the LP group. Therefore, dexmedetomidine enhanced mt16S expression (Figure 4).

### Dexmedetomidine improves structural changes in the kidneys of mice with SAKI

**Figure 5.**
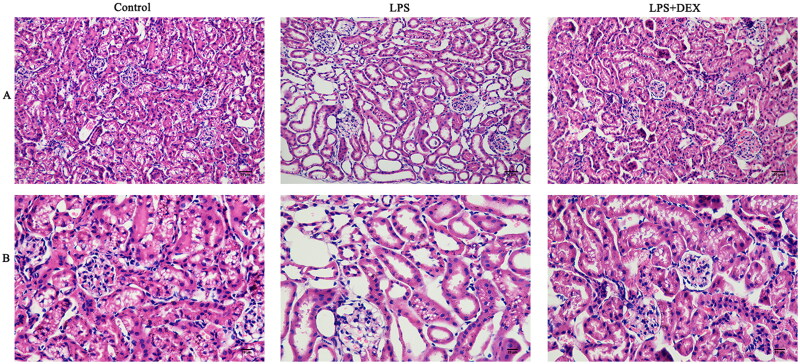
Dexmedetomidine improves renal structural changes in mice with SAKI. A: Image showing renal structure using an optical microscope at 200× magnification; B: Image showing renal structure using an optical microscope at 400× magnification. Mouse kidney tissues were subjected to hematoxylin (blue) for staining cell nuclei and eosin (orange-red) for staining cytoplasm.

Hematoxylin and eosin staining of the kidney tissues revealed more disorganized kidney structures in the LPS groups than in the control group. In contrast, kidney tissues in the LPS+DEX group exhibited normal morphology. These findings indicated that dexmedetomidine could mitigate structural alterations in the kidneys associated with SAKI (Figure 5).

### Dexmedetomidine regulates Pink1/Park2/Optineurin expression to improve SAKI

**Figure 6.**
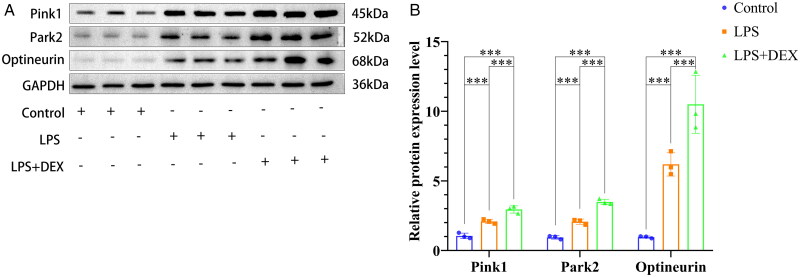
Dexmedetomidine improves SAKI by upregulating the Pink1/Park2/optineurin mitophagy pathway. A: Western blotting analysis of the expression levels of autophagy-related proteins Pink1, Park2, and optineurin in kidney tissues. B: ***indicates *p* < 0.001.

Western blotting was employed to evaluate the effect of dexmedetomidine on Pink1/Park2/Optineurin autophagy pathway. Compared with the control group, both the LPS and LPS+DEX groups exhibited upregulated Pink1, Park2, and Optineurin expression. Additionally, their expressions were further upregulated in the LPS+DEX group compared with those in the LPS group. Therefore, dexmedetomidine improved SAKI by regulating the Pink1/Park2/Optineurin autophagy pathway (Figure 6).

### Dexmedetomidine reduces apoptosis in mice with SAKI

**Figure 7.**
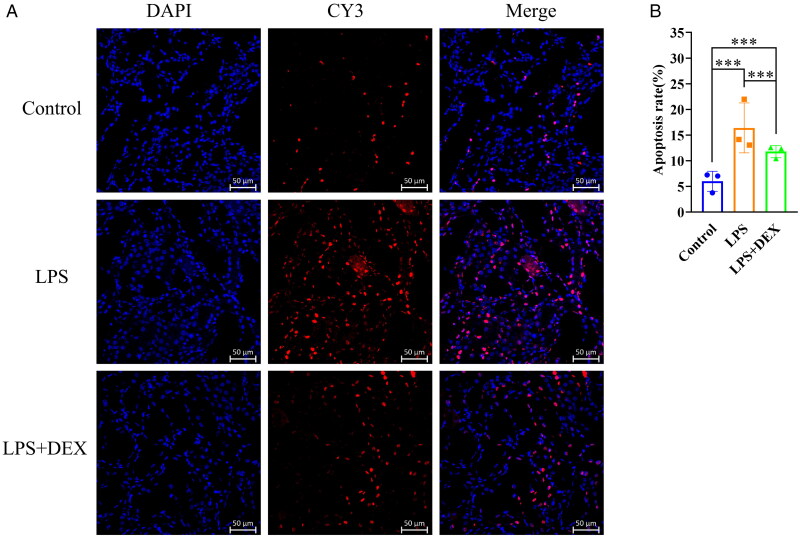
Dexmedetomidine reduces apoptosis of renal tissue cells in mice with SAKI. A: In the image, red cells represent apoptotic cells, and blue cells represent normal cells. B: *** indicates a highly significant difference between the two groups (*p* < 0.001); *Indicates a significant difference between the two groups (*p* < 0.05).

The apoptosis rate of renal tissue cells in both the LPS and LPS+DEX groups was significantly higher than that in the control group. Furthermore, compared with the LPS group, the LPS+DEX group exhibited significantly decreased apoptosis rate in renal tissue cells. Therefore, dexmedetomidine reduced the apoptotic rate of renal tissue cells (Figure 7).

## Discussion

SAKI exhibits a high incidence and significantly affects patient prognosis with high mortality rates [15]. Currently, effective preventive or therapeutic measures are unavailable for SAKI. Dexmedetomidine, one of the most commonly used sedatives in the ICU, has garnered increasing attention for its potential role in SAKI [16]. Our results demonstrated that LPS can induce SAKI, leading to elevated levels of various inflammatory factors, including TNF-α, IL-18, IL-6, and IL-1β. However, pretreatment with dexmedetomidine significantly reduced their levels in the mouse model of SAKI. This finding is consistent with those of previous studies. For example, Zhang et al. found that dexmedetomidine could inhibit the release of TNF-α and IL-1β in septic mice, thereby alleviating the inflammatory response and organ damage [17]. Similarly, Tan et al. reported reduced IL-6 and IL-8 levels in the serum of septic rats injected with LPS following dexmedetomidine treatment [18]. The results of the present study provide new experimental evidence of the anti-inflammatory effects of dexmedetomidine in SAKI.

Following LPS administration, the mice exhibited reduced kidney function, and histopathological examination revealed significant kidney tissue damage. Dexmedetomidine reduced the levels of several kidney function-related biomarkers, including Cys-C, Cr, BUN, urinary KIM1, and NGAL, indicating improvement in kidney function [19,20]. Additionally, the dexmedetomidine-treated group demonstrated more normal kidney tissue structure, further supporting its protective effects. These findings demonstrate the protective role of dexmedetomidine against SAKI from the perspective of both biochemical markers and tissue morphology. This observation is consistent with findings from other studies. For example, Chen et al. reported improvement in SAKI following dexmedetomidine use through animal experiments [21]. Furthermore, Hu et al. analyzed data from 2,192 critically ill patients with SAKI in the MMIC database and found that dexmedetomidine administration was associated with improved renal recovery and increased in-hospital survival rates in these patients [22].

Although the increase in serum Cr observed in our study was close to but slightly below a two-fold change compared with the control group, the LPS-induced model still met the accepted diagnostic criteria for AKI in mice. According to modified KDIGO-based definitions applied in murine models [23,24], AKI is characterized by increases in serum creatinine and/or BUN exceeding 1.5-fold over baseline, along with evidence of tubular injury. In our study, BUN increased by more than 1.5-fold, and additional renal injury biomarkers, including KIM1, NGAL, and Cys-C, were also markedly elevated. These biochemical changes were supported by consistent histopathological findings, including tubular damage and increased apoptosis. Taken together, these results confirm the successful establishment of a SAKI model in mice without the need for further validation.

Mitochondria are targets of cellular oxidative stress, and mitochondrial dysfunction is implicated in the pathophysiology of SAKI [25]. The intraperitoneal injection of LPS induced SAKI, leading to a redox imbalance in the renal tissue in this study. Notably, dexmedetomidine could effectively maintain the cellular redox balance and improve mitochondrial function, thereby protecting against SAKI. This finding was primarily reflected in several aspects. First, dexmedetomidine reduced ROS and MDA levels. Mitochondria are the main source of ROS within cells, and damage to mitochondria may result in ROS production in large amounts. Excessive ROS production subsequently causes oxidative stress and tissue damage [26]. MDA is a product of lipid peroxidation, and its levels reflect the degree of oxidative stress in the body [27]. The reduction in MDA levels indicated that dexmedetomidine could effectively inhibit oxidative stress, thereby mitigating renal cell damage. Second, dexmedetomidine decreased CAT and GSH production. GSH and CAT play crucial roles in maintaining the cellular redox balance [28], and their reduced levels may reflect an improvement in the antioxidant defense system. Furthermore, SOD is an important antioxidant enzyme, and enhancing SOD activity can reduce mitochondrial oxidative stress [29]. Dexmedetomidine also increased SOD activity, thereby enhancing the antioxidant capacity of cells and inhibiting mitochondrial oxidative damage. Finally, dexmedetomidine increased mt16S expression, which may be related to the improvement in mitochondrial function. These findings are consistent with those of previous studies. For example, Kiyonaga et al. reported improvement in mitochondrial dysfunction following dexmedetomidine treatment along with its protective effect on the kidneys [30]. Furthermore, Chen et al. reported that dexmedetomidine reduced oxidative stress and improved kidney function [31].

Mitophagy is an important cellular quality control mechanism that selectively eliminates damaged mitochondria, thereby maintaining cellular homeostasis [10]. The Pink1/Park2 pathway is the most extensively studied mitophagy pathway in mammals [32]. In healthy mitochondria, transport proteins on the inner and outer mitochondrial membranes work together to translocate Pink1 from the cytoplasm to the mitochondria, where it is cleaved by presenilin-associated rhomboid-like protein. This cleavage leads to rapid degradation of Pink1 by the ubiquitin-proteasome system [33]. However, when mitochondria are damaged, Pink1 accumulates in the outer mitochondrial membrane. Following its phosphorylation, Pink1 activates Park2, and phosphorylated Park2 conjugates ubiquitin to various proteins on the mitochondrial outer membrane. The resulting ubiquitin chains are recruited to damaged mitochondria and bind to autophagy receptors, thereby mediating mitophagy to remove damaged mitochondria [34]. Multiple studies have shown that mitophagy facilitates the recovery from acute kidney injury. For example, Liu et al. demonstrated the protective effect of Pink1/Park2-dependent mitophagy against NaAsO_2_-induced acute kidney injury [35]. Furthermore, Zhao et al. discovered that inhibition of the Pink1/Park2 mitophagy pathway worsens renal ischemia-reperfusion injury [36]. Sabry et al. reported that resveratrol elevated mitochondrial Pink1/Park2 levels, reduced renal angiotensin levels, and exerted beneficial effects in acute kidney injury [37]. However, to date, research on whether dexmedetomidine protects against SAKI by regulating the Pink1/Park2-dependent mitophagy pathway remains insufficient.

The present study showed that dexmedetomidine increased the expression of the mitophagy-related proteins Pink1, Park2, and Optineurin, while immunofluorescence analysis demonstrated a significant reduction in renal cell apoptosis following treatment. These findings suggest that dexmedetomidine may exert its renoprotective effects by activating the Pink1/Park2/Optineurin mitophagy pathway, thereby promoting mitophagy, limiting mitochondrial damage, attenuating oxidative stress, enhancing cellular energy metabolism, and reducing apoptosis. In addition to its effects on mitophagy, dexmedetomidine has also been reported to improve renal outcomes *via* hemodynamic and tubular mechanisms [38]. For instance, studies in ovine septic shock models have shown that dexmedetomidine enhances renal medullary perfusion through local vasodilation, increases glomerular filtration, reduces arginine vasopressin activity in the collecting duct, and downregulates aquaporin expression and sodium-water transport [39,40]. Moreover, dexmedetomidine can reduce sympathetic tone, stabilize renal perfusion pressure and decrease circulating catecholamines [41]. These mechanisms may contribute to improved renal function independently of autophagy and these effects could indirectly contribute to the observed outcomes, making it challenging to isolate the specific role of mitophagy-dependent mechanisms without further comparative studies or real-time hemodynamic monitoring. Thus, future studies comparing dexmedetomidine with other sedatives and incorporating direct hemodynamic monitoring could help clarify the specificity of the observed effects and provide a more comprehensive understanding of its mechanisms.

Our study also focused on mitophagy, which may also underlie the observed protective effects and warrant further exploration. Moreover, sepsis pathophysiology involves additional contributors, including histone release and endothelial dysfunction, which interact with inflammatory and oxidative processes to mediate renal injury [42]. It is also important to note that despite the LPS-induced SAKI model used in this study could replicate key pathological features of SAKI, murine LPS models typically induce a hypodynamic circulatory state, whereas human sepsis often presents as a hyperdynamic condition, as well as observed in larger animal models such as pigs or sheep [43,44]. Moreover, no volume resuscitation was administered in our protocol, which may limit the relevance of hemodynamic responses and renal perfusion compared with clinical practice. These differences can impact the interpretation of drug effects on inflammation, oxidative stress, and renal function. While rodent models are valuable for mechanistic investigations, further validation using large-animal models that incorporate fluid management and replicate human cardiovascular physiology will be essential to support clinical translation of these findings.

Despite the interesting findings reported, this study has several limitations. First, the small sample size (three mice per group), due to limited funding, reduced the statistical power and precluded formal testing of data normality. Although nonparametric tests were considered, their limited sensitivity in small datasets resulted in loss of significance for several comparisons. Therefore, we retained t-tests for consistency. Given these constraints, the findings should be interpreted as preliminary and hypothesis-generating. Second, although our data demonstrate a consistent association between dexmedetomidine treatment and activation of the Pink1/Park2 mitophagy pathway, no functional inhibition studies (e.g., siRNA knockdown or pharmacological blockade) were performed to establish causality. This mechanistic validation will be a key focus of future investigations. Third, volume resuscitation was not administered, which may limit clinical relevance given its importance in sepsis management. Fourth, the renoprotective effects of dexmedetomidine may also involve sedative- or hemodynamic-related mechanisms, which were not controlled for in this study and may confound interpretation of autophagy-related findings. Lastly, other mitochondrial regulatory processes such as fusion, fission, and biogenesis were not evaluated. These limitations will be addressed in future studies employing larger sample sizes, functional assays, and expanded mechanistic analyses to validate and extend the current findings.

## Conclusions

In conclusion, this preliminary study suggests that dexmedetomidine may confer protection against SAKI by modulating the Pink1/Park2 mitophagy pathway and reducing oxidative stress and apoptosis. These findings provide exploratory insights into the potential utility of dexmedetomidine in SAKI management and highlight avenues for future mechanistic and translational research.

## Data Availability

The analyzed data sets generated during the study are available from the corresponding author on reasonable request.
